# Supplementary figures and images for: Are consumer confidence and asset value expectations positively associated with length of daylight?: An exploration of psychological mediators between length of daylight and seasonal asset price transitions
Source: PLoS One. 2021 Jan 20;16(1):e0245520. doi: 10.1371/journal.pone.0245520 (PMC7817041; doi:10.1371/journal.pone.0245520)

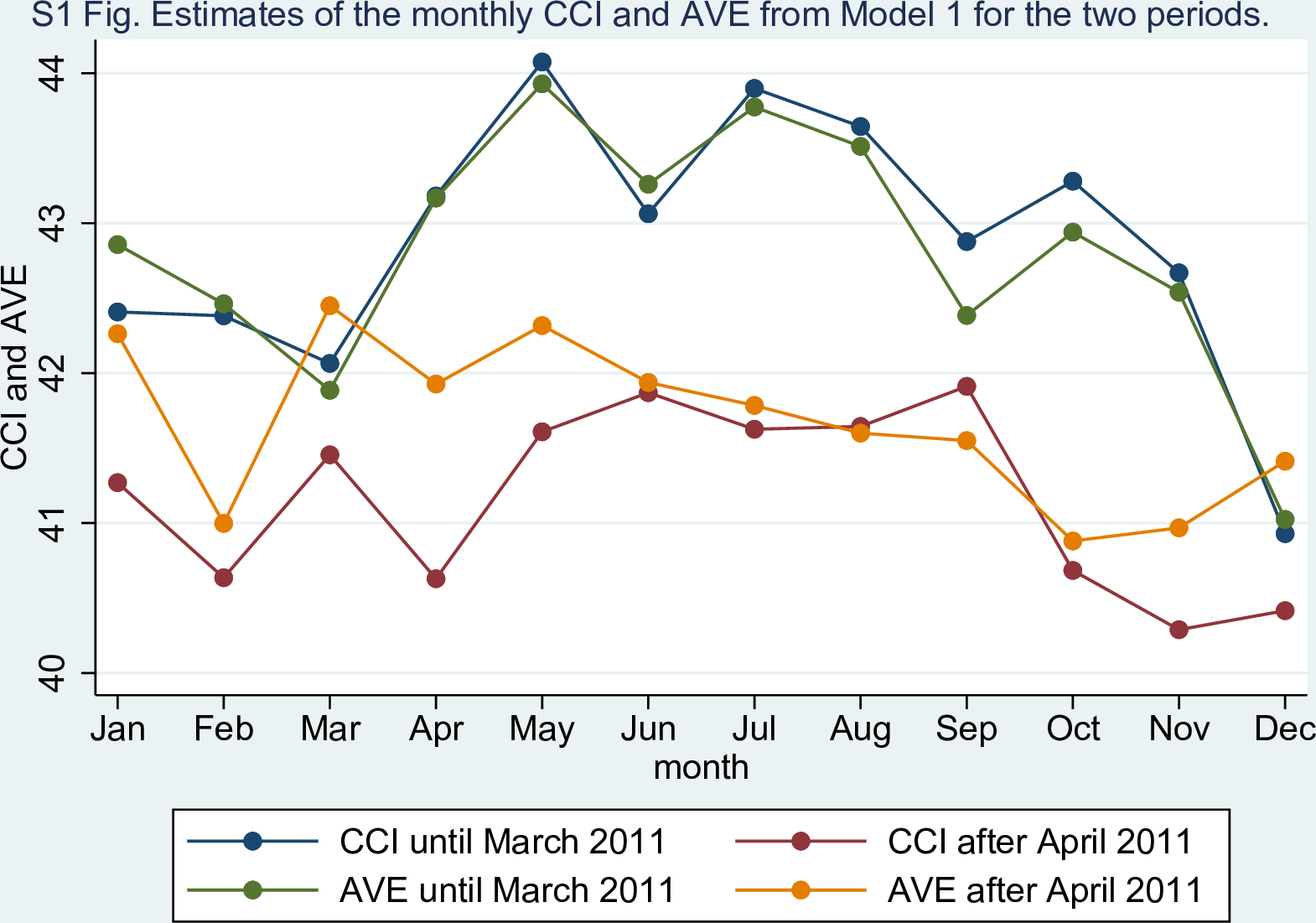

Supplement: S1 Fig — (TIF) [file pone.0245520.s001.tif]

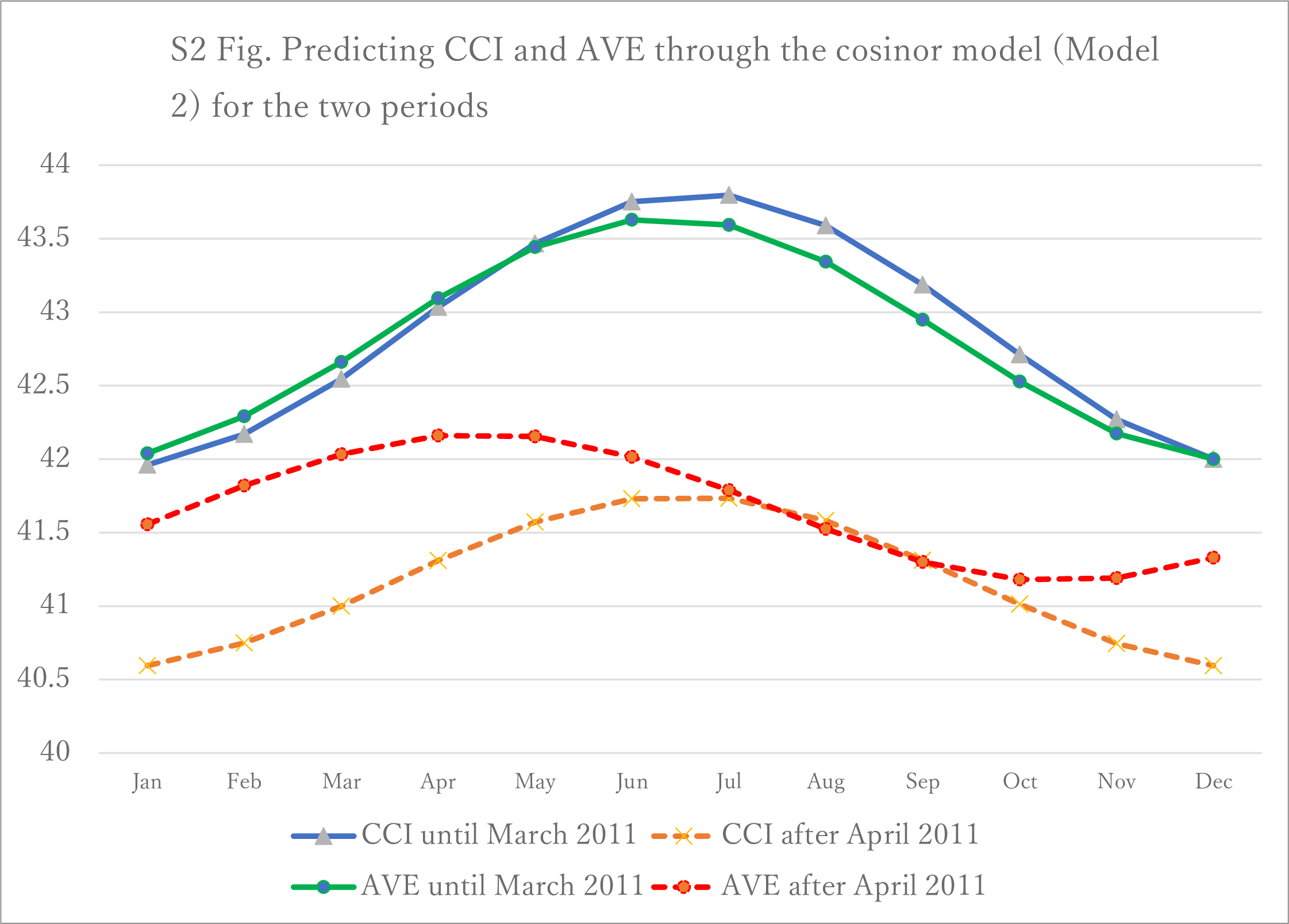

Supplement: S2 Fig — (TIF) [file pone.0245520.s002.tif]

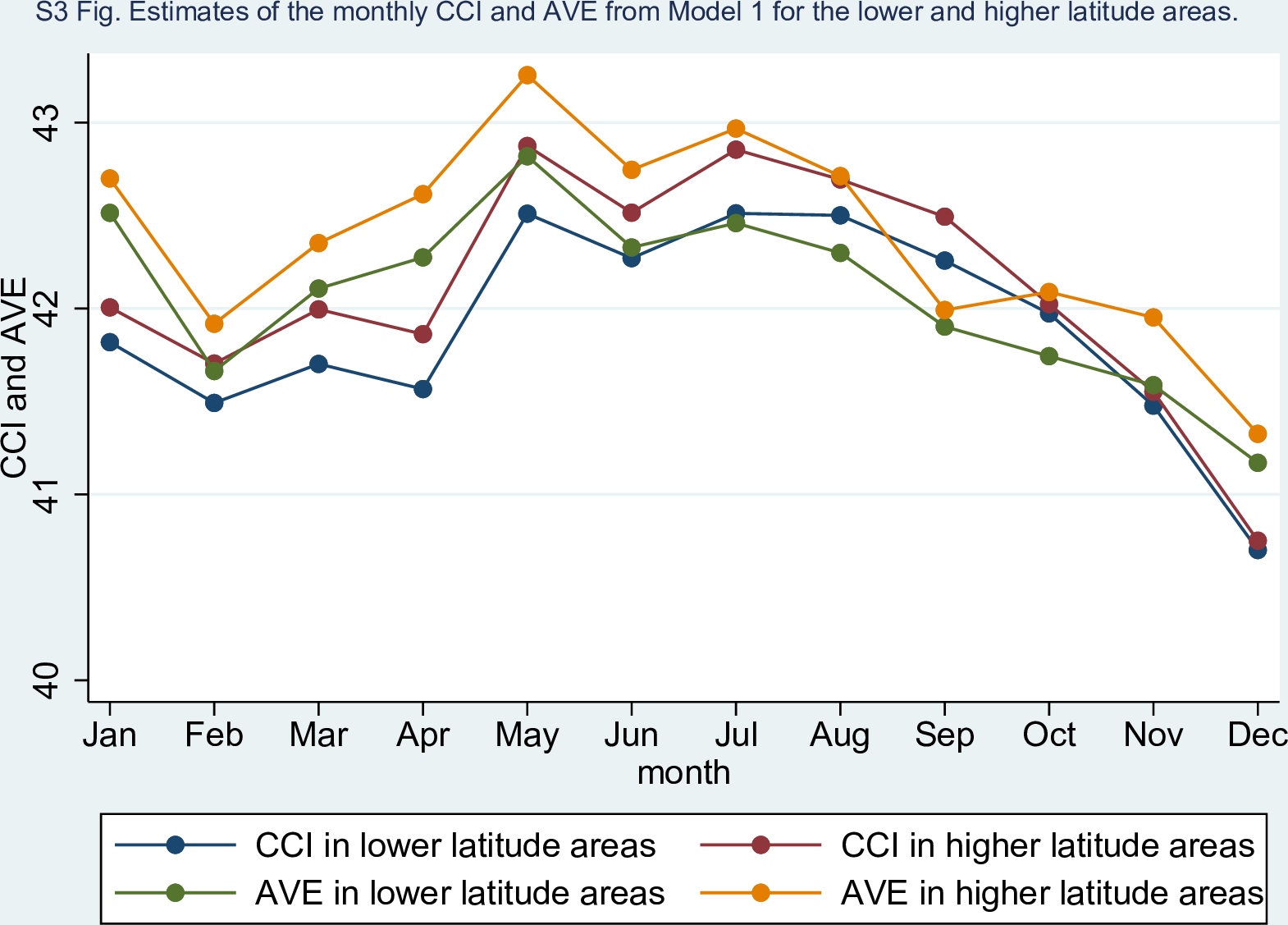

Supplement: S3 Fig — (TIF) [file pone.0245520.s003.tif]

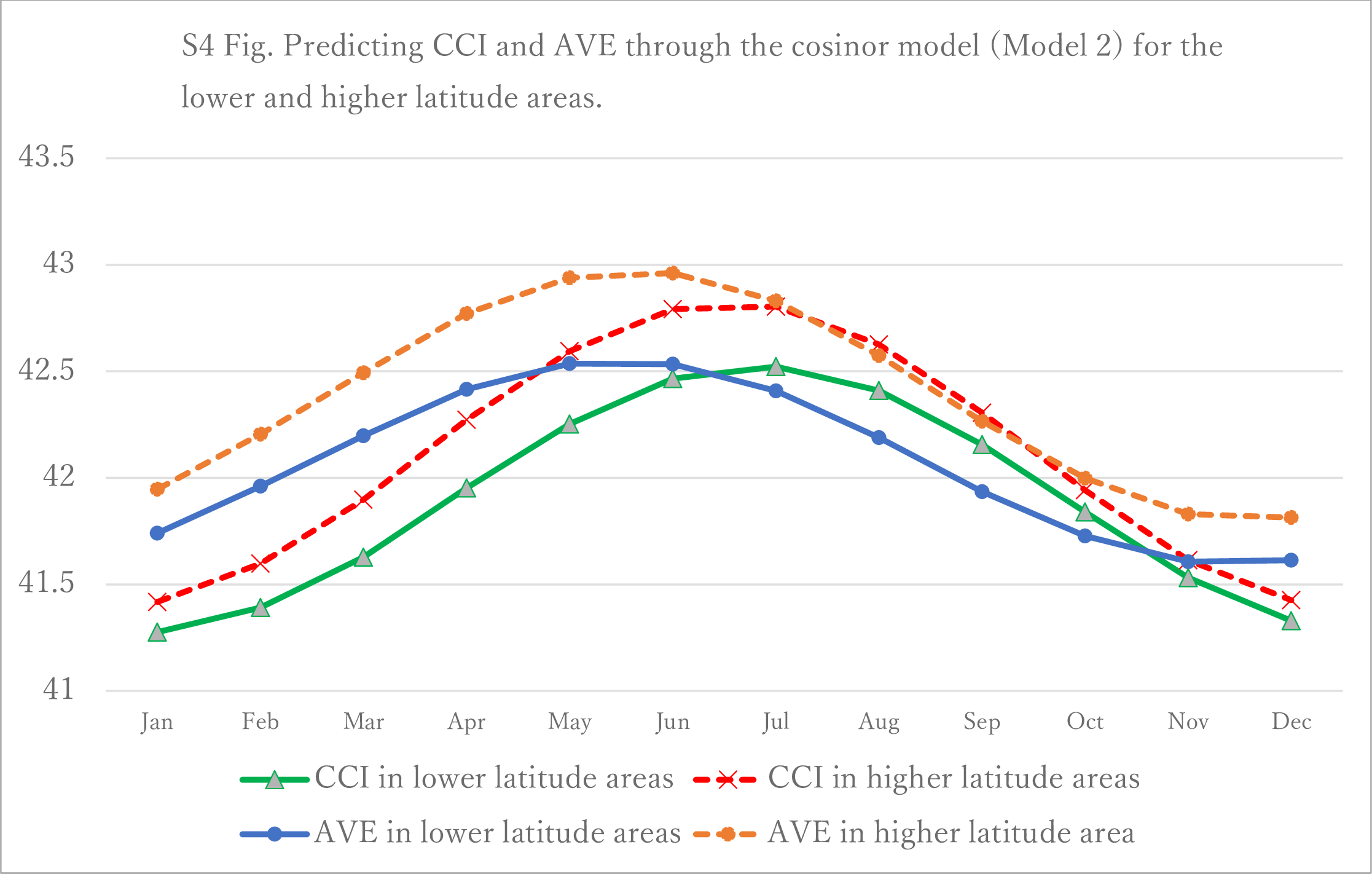

Supplement: S4 Fig — (TIF) [file pone.0245520.s004.tif]
