# Supplementary material for: Are consumer confidence and asset value expectations positively associated with length of daylight?: An exploration of psychological mediators between length of daylight and seasonal asset price transitions
Source: PLoS One. 2021 Jan 20;16(1):e0245520. doi: 10.1371/journal.pone.0245520 (PMC7817041; doi:10.1371/journal.pone.0245520)
Supplement: S1 Table — (DOCX) [file pone.0245520.s005.docx]

**S1 Table**

*Summary Statistics of Participating Households from the Consumer Confidence Survey (CCS)*

| Prefecture | Number  of observations | Number of unique households | Mean age of household  head | Ratio of female household heads | Ratio of household heads in the labor force | Mean of CCI  (SD) | | Mean of AVE  (SD) | |
| --- | --- | --- | --- | --- | --- | --- | --- | --- | --- |
| Hokkaido | 44,504 | 3,951 | 58.6 | 23.5% | 60.5% | 39.83 | (15.18) | 40.94 | (17.76) |
| Aomori | 10,740 | 903 | 58.7 | 23.7% | 62.3% | 40.85 | (14.74) | 41.43 | (17.15) |
| Iwate | 10,278 | 882 | 58.4 | 24.7% | 63.5% | 40.93 | (15.25) | 41.53 | (17.53) |
| Miyagi | 17,121 | 1,596 | 58.0 | 23.0% | 63.8% | 41.55 | (14.79) | 42.90 | (17.54) |
| Akita | 10,241 | 922 | 61.2 | 24.5% | 54.1% | 40.72 | (15.35) | 39.92 | (18.15) |
| Yamagata | 10,554 | 971 | 63.1 | 23.7% | 57.4% | 40.19 | (15.19) | 40.38 | (18.46) |
| Fukushima | 16,219 | 1,443 | 61.3 | 22.1% | 60.6% | 40.71 | (15.01) | 40.83 | (18.29) |
| Ibaraki | 19,665 | 1,821 | 59.9 | 22.0% | 63.5% | 41.54 | (14.58) | 41.11 | (17.81) |
| Tochigi | 14,449 | 1,254 | 59.1 | 21.1% | 64.9% | 42.48 | (14.63) | 41.87 | (17.73) |
| Gunma | 14,103 | 1,210 | 61.7 | 24.3% | 56.9% | 42.72 | (13.65) | 42.62 | (17.11) |
| Saitama | 44,737 | 3,931 | 57.5 | 20.3% | 67.1% | 42.05 | (15.13) | 42.19 | (18.33) |
| Chiba | 40,586 | 3,645 | 59.9 | 20.2% | 59.7% | 43.31 | (14.01) | 42.55 | (18.30) |
| Tokyo | 93,763 | 8,611 | 57.5 | 22.4% | 70.1% | 43.17 | (15.14) | 44.34 | (18.86) |
| Kanagawa | 59,326 | 5,271 | 57.8 | 20.3% | 65.5% | 42.82 | (14.93) | 43.39 | (18.36) |
| Niigata | 16,236 | 1,431 | 58.4 | 20.8% | 63.9% | 42.21 | (14.98) | 41.98 | (17.61) |
| Toyama | 9,527 | 780 | 60.2 | 19.4% | 63.8% | 43.19 | (14.02) | 42.22 | (17.82) |
| Ishikawa | 10,332 | 880 | 60.2 | 20.9% | 63.0% | 42.80 | (13.46) | 42.80 | (16.26) |
| Fukui | 9,474 | 824 | 59.4 | 22.6% | 66.0% | 42.09 | (14.59) | 41.67 | (17.94) |
| Yamanashi | 9,166 | 819 | 61.5 | 22.7% | 63.9% | 41.59 | (15.18) | 40.10 | (18.62) |
| Nagano | 16,201 | 1,434 | 59.5 | 20.9% | 68.4% | 42.55 | (15.37) | 42.00 | (18.16) |
| Gifu | 14,360 | 1,233 | 60.6 | 21.8% | 62.3% | 41.50 | (14.59) | 41.02 | (18.08) |
| Shizuoka | 26,044 | 2,283 | 59.6 | 22.1% | 63.6% | 41.83 | (15.07) | 42.02 | (18.05) |
| Aichi | 49,732 | 4,382 | 58.9 | 20.3% | 64.9% | 42.94 | (14.41) | 43.82 | (17.32) |
| Mie | 14,558 | 1,226 | 62.2 | 25.0% | 57.8% | 43.41 | (13.64) | 42.87 | (17.20) |
| Shiga | 10,474 | 862 | 58.8 | 22.6% | 60.0% | 41.95 | (14.68) | 41.11 | (18.17) |
| Kyoto | 22,126 | 1,951 | 61.5 | 22.7% | 61.0% | 41.75 | (14.78) | 42.20 | (18.28) |
| Osaka | 62,676 | 5,670 | 60.2 | 24.0% | 60.3% | 41.54 | (15.03) | 41.90 | (18.19) |
| Hyogo | 39,751 | 3,547 | 60.9 | 22.8% | 56.3% | 41.96 | (14.72) | 41.84 | (18.36) |
| Nara | 10,349 | 905 | 58.9 | 24.2% | 60.3% | 42.25 | (14.53) | 40.65 | (18.95) |
| Wakayama | 10,332 | 876 | 63.6 | 27.4% | 57.4% | 40.27 | (15.29) | 40.15 | (18.69) |
| Tottori | 9,225 | 761 | 61.2 | 24.0% | 56.8% | 42.03 | (14.55) | 41.56 | (17.62) |
| Shimane | 9,345 | 782 | 58.7 | 19.6% | 63.7% | 41.88 | (14.44) | 42.30 | (17.36) |
| Okayama | 16,340 | 1,402 | 59.2 | 23.2% | 64.8% | 41.55 | (14.60) | 41.48 | (18.12) |
| Hiroshima | 23,128 | 2,068 | 58.0 | 20.8% | 64.3% | 42.24 | (14.50) | 42.15 | (17.44) |
| Yamaguchi | 13,429 | 1,162 | 59.5 | 23.6% | 62.7% | 42.35 | (14.67) | 41.54 | (18.12) |
| Tokushima | 9,258 | 762 | 59.8 | 24.1% | 66.9% | 40.93 | (15.28) | 39.71 | (19.34) |
| Kagawa | 10,431 | 897 | 59.8 | 23.5% | 62.4% | 42.32 | (14.05) | 40.89 | (18.49) |
| Ehime | 11,445 | 994 | 58.8 | 23.3% | 63.5% | 42.32 | (14.54) | 41.78 | (18.13) |
| Kochi | 9,127 | 794 | 58.2 | 24.8% | 62.3% | 39.56 | (15.77) | 40.96 | (18.40) |
| Fukuoka | 36,001 | 3,408 | 58.1 | 23.7% | 63.2% | 41.03 | (15.52) | 42.03 | (18.09) |
| Saga | 9,137 | 793 | 60.1 | 27.1% | 61.7% | 41.53 | (14.58) | 41.61 | (17.21) |
| Nagasaki | 11,443 | 1,041 | 60.2 | 24.7% | 61.6% | 40.41 | (14.59) | 41.51 | (16.88) |
| Kumamoto | 12,864 | 1,161 | 59.8 | 23.8% | 61.6% | 42.40 | (14.60) | 43.13 | (17.06) |
| Oita | 10,174 | 935 | 58.1 | 23.5% | 65.7% | 42.90 | (14.17) | 42.37 | (16.82) |
| Miyazaki | 9,764 | 885 | 61.3 | 28.1% | 57.2% | 41.13 | (15.33) | 41.32 | (17.82) |
| Kagoshima | 15,909 | 1,462 | 57.9 | 21.1% | 67.1% | 42.32 | (15.21) | 41.74 | (18.30) |
| Okinawa | 9,717 | 932 | 55.7 | 29.5% | 66.5% | 43.32 | (15.16) | 46.21 | (15.29) |
| Total | 964,361 | 85,753 | 59.2 | 22.5% | 63.2% | 42.02 | (14.86) | 42.24 | (18.07) |

SD = Standard Deviation, CCI = Consumer Confidence Index, AVE = Asset Value Expectation.
