# Supplementary material for: Are consumer confidence and asset value expectations positively associated with length of daylight?: An exploration of psychological mediators between length of daylight and seasonal asset price transitions
Source: PLoS One. 2021 Jan 20;16(1):e0245520. doi: 10.1371/journal.pone.0245520 (PMC7817041; doi:10.1371/journal.pone.0245520)
Supplement: S2 Table — (DOCX) [file pone.0245520.s006.docx]

| **S2 Table. The monthly summary statistics of CCI and AVE for the two periods**.  Monthly statistics of CCI and AVE from April 2004 to August 2018. | | | | | | | | | | |  |  |
| --- | --- | --- | --- | --- | --- | --- | --- | --- | --- | --- | --- | --- |
|  | CCI until March 2011 | | | CCI after April 2011 | | | AVE until March 2011 | | | AVE after April 2011 | | |
|  | n | Mean | SD | n | Mean | SD | n | Mean | SD | n | Mean | SD |
| January | 39,009 | 42.24 | (15.14) | 38,202 | 41.43 | (15.06) | 39,009 | 42.67 | (17.67) | 38,233 | 42.41 | (18.29) |
| February | 38,990 | 42.29 | (15.16) | 38,414 | 40.92 | (15.07) | 38,990 | 42.35 | (17.63) | 38,440 | 41.25 | (18.41) |
| March | 38,625 | 41.52 | (15.48) | 38,306 | 41.79 | (14.92) | 38,625 | 41.36 | (20.17) | 38,336 | 42.75 | (18.12) |
| April | 40,269 | 43.56 | (14.72) | 44,658 | 40.48 | (15.20) | 40,269 | 43.57 | (17.49) | 44,738 | 41.83 | (18.47) |
| May | 40,223 | 44.46 | (14.08) | 44,730 | 41.53 | (14.89) | 40,223 | 44.32 | (16.27) | 44,783 | 42.25 | (18.18) |
| June | 39,622 | 43.11 | (14.73) | 44,530 | 41.82 | (14.69) | 39,622 | 43.36 | (19.35) | 44,583 | 41.89 | (17.91) |
| July | 39,557 | 44.10 | (14.09) | 44,686 | 41.63 | (14.67) | 39,557 | 43.95 | (16.15) | 44,708 | 41.77 | (17.91) |
| August | 39,557 | 43.85 | (14.13) | 44,398 | 41.66 | (14.48) | 39,557 | 43.67 | (16.37) | 44,421 | 41.56 | (17.65) |
| September | 39,287 | 42.77 | (14.53) | 38,332 | 41.65 | (14.69) | 39,287 | 42.31 | (18.96) | 38,363 | 41.34 | (18.08) |
| October | 39,283 | 43.22 | (14.18) | 38,525 | 40.52 | (14.84) | 39,283 | 42.87 | (17.19) | 38,558 | 40.75 | (17.94) |
| November | 39,281 | 42.67 | (14.69) | 38,320 | 40.20 | (15.03) | 39,281 | 42.51 | (17.40) | 38,343 | 40.89 | (18.33) |
| December | 39,024 | 40.65 | (15.69) | 38,074 | 40.46 | (15.23) | 39,024 | 40.76 | (19.98) | 38,089 | 41.43 | (18.54) |
| SD = Standard Deviation, CCI = Consumer Confidence Index, AVE = Asset Value Expectation | | | | | | | | | | | | |
