# Supplementary material for: Are consumer confidence and asset value expectations positively associated with length of daylight?: An exploration of psychological mediators between length of daylight and seasonal asset price transitions
Source: PLoS One. 2021 Jan 20;16(1):e0245520. doi: 10.1371/journal.pone.0245520 (PMC7817041; doi:10.1371/journal.pone.0245520)
Supplement: S3 Table — (DOCX) [file pone.0245520.s007.docx]

| **S3 Table. Fixed-effects model estimation of monthly CCI and AVE from Model 1 for the two periods**. | | | | | | |  | |  | |
| --- | --- | --- | --- | --- | --- | --- | --- | --- | --- | --- |
|  | CCI until March 2011 | | CCI after April 2011 | | AVE until March 2011 | | | AVE after April 2011 | | |
| 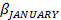   \|  \| \| --- \| | 1.478*** | (0.062) | 0.854*** | (0.059) | 1.833*** | (0.102) | | 0.850*** | | (0.091) |
| 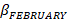   \|  \| \| --- \| | 1.453*** | (0.066) | 0.220*** | (0.064) | 1.438*** | (0.103) | | -0.415*** | | (0.097) |
| 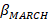   \|  \| \| --- \| | 1.135*** | (0.071) | 1.039*** | (0.067) | 0.861*** | (0.109) | | 1.037*** | | (0.097) |
| 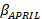   \|  \| \| --- \| | 2.252*** | (0.074) | 0.213** | (0.068) | 2.143*** | (0.110) | | 0.514*** | | (0.098) |
| 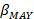   \|  \| \| --- \| | 3.146*** | (0.074) | 1.193*** | (0.067) | 2.905*** | (0.109) | | 0.905*** | | (0.095) |
| 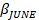   \|  \| \| --- \| | 2.133*** | (0.076) | 1.454*** | (0.067) | 2.235*** | (0.114) | | 0.525*** | | (0.094) |
| 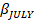   \|  \| \| --- \| | 2.969*** | (0.074) | 1.210*** | (0.067) | 2.750*** | (0.109) | | 0.372*** | | (0.094) |
| 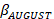   \|  \| \| --- \| | 2.715*** | (0.073) | 1.228*** | (0.065) | 2.487*** | (0.108) | | 0.187* | | (0.092) |
| 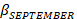   \|  \| \| --- \| | 1.948*** | (0.071) | 1.496*** | (0.065) | 1.360*** | (0.108) | | 0.137 | | (0.094) |
| 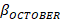   \|  \| \| --- \| | 2.350*** | (0.068) | 0.268*** | (0.062) | 1.916*** | (0.105) | | -0.532*** | | (0.092) |
| 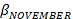   \|  \| \| --- \| | 1.739*** | (0.064) | -0.127* | (0.058) | 1.515*** | (0.103) | | -0.444*** | | (0.089) |
| Intercept | 40.930*** | (0.049) | 40.416*** | (0.045) | 41.025*** | (0.077) | | 41.413*** | | (0.066) |
| No. of observations | 472,727 | | 491,175 | | 472,727 | | | 491,595 | | |
| No. of groups | 44,654 | | 45,636 | | 44,654 | | | 45,649 | | |
| R-squared (within) | 0.008 | | 0.004 | | 0.003 | | | 0.002 | | |
| R-squared (between) | 0.015 | | 0.000 | | 0.013 | | | 0.000 | | |
| R-squared (overall) | 0.005 | | 0.001 | | 0.003 | | | 0.001 | | |
| CCI = Consumer Confidence Index, AVE = Asset Value Expectation. * *p* < 5%, ** *p* < 1%, *** *p* < 0.1%. Robust standard errors are in parentheses. CCI and AVE were indexed based on the formula from the Cabinet Office of Japan. | | | | | | | | | | |
