# Supplementary material for: Are consumer confidence and asset value expectations positively associated with length of daylight?: An exploration of psychological mediators between length of daylight and seasonal asset price transitions
Source: PLoS One. 2021 Jan 20;16(1):e0245520. doi: 10.1371/journal.pone.0245520 (PMC7817041; doi:10.1371/journal.pone.0245520)
Supplement: S4 Table — (DOCX) [file pone.0245520.s008.docx]

| **S4 Table. Fixed-effects model estimation of CCI and AVE through cosinor model (Model 2) with amplitude and acrophase for the two periods.** | | | | | | | | |
| --- | --- | --- | --- | --- | --- | --- | --- | --- |
|  | CCI until March 2011 | | CCI after April 2011 | | AVE until March 2011 | | AVE after April 2011 | |
| 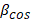 | -0.929*** | (0.028) | -0.588*** | (0.025) | -0.821*** | (0.035) | -0.229*** | (0.031) |
| 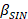 | -0.063* | (0.026) | 0.0090 | (0.025) | 0.090** | (0.033) | 0.448*** | (0.033) |
| Intercept | 42.877*** | (0.000) | 41.164*** | (0.001) | 42.815*** | (0.000) | 41.671*** | (0.002) |
| No. of observations | 472,727 | | 491,175 | | 472,727 | | 491,595 | |
| No. of groups | 44,654 | | 45,636 | | 44,654 | | 45,649 | |
| R-squared (within) | 0.005 | | 0.002 | | 0.002 | | 0.001 | |
| R-squared (between) | 0.010 | | 0.000 | | 0.010 | | 0.000 | |
| R-squared (overall) | 0.003 | | 0.001 | | 0.002 | | 0.000 | |
| Amplitude | 0.931 | (0.028) | 0.588 | (0.025) | 0.826 | (0.035) | 0.503 | (0.033) |
| Acrophase | 187.408 | (1.642) | 182.615 | (2.465) | 177.148 | (2.334) | 119.638 | (3.634) |
| CCI = Consumer Confidence Index, AVE = Asset Value Expectation. * *p* < 5%, ** *p* < 1%, *** *p* < 0.1%. Robust standard errors are in parentheses except for amplitude and acrophase. For these, standard errors were calculated using the delta method. CCI and AVE were indexed based on the formula from the Cabinet Office of Japan. | | | | | | | | |
