# Supplementary material for: Are consumer confidence and asset value expectations positively associated with length of daylight?: An exploration of psychological mediators between length of daylight and seasonal asset price transitions
Source: PLoS One. 2021 Jan 20;16(1):e0245520. doi: 10.1371/journal.pone.0245520 (PMC7817041; doi:10.1371/journal.pone.0245520)
Supplement: S5 Table — (DOCX) [file pone.0245520.s009.docx]

| **S5 Table. Fixed-effects model estimation of CCI with length of daylight, cloud cover, precipitation, and temperature (Model 3) for the two periods.** | | | | | | | | |
| --- | --- | --- | --- | --- | --- | --- | --- | --- |
|  | CCI until March 2011 | | CCI until March 2011 | | CCI after April 2011 | | CCI after April 2011 | |
| 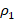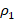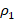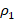   \|  \| \| --- \| | 0.193*** | (0.003) | 0.193*** | (0.003) | 0.196*** | (0.003) | 0.196*** | (0.003) |
| 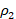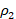   \|  \| \| --- \| | 0.058*** | (0.002) | 0.058*** | (0.002) | 0.054*** | (0.002) | 0.054*** | (0.002) |
| (per hour)*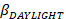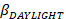* | 0.313*** | (0.010) | 0.273*** | (0.013) | 0.210*** | (0.009) | 0.179*** | (0.013) |
| (per one point)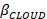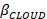   \| (per hour) \| \| --- \| |  |  | 0.042*** | (0.010) |  |  | 0.054*** | (0.008) |
| *(per 1mm/day)*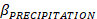   \| *(per 1mm/day)* \| \| --- \| |  |  | 0.001** | (0.001) |  |  | -0.003*** | (0.001) |
| (per ℃)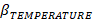   \| (per ℃) \| \| --- \| |  |  | 0.008** | (0.003) |  |  | 0.005 | (0.003) |
| Intercept | 28.315*** | (0.199) | 28.388*** | (0.213) | 28.275*** | (0.177) | 28.287*** | (0.200) |
| No. of observations | 385,073 | | 385,073 | | 392,440 | | 392,440 | |
| No. of groups | 39,395 | | 39,395 | | 40,077 | | 40,077 | |
| R-squared (within) | 0.052 | | 0.052 | | 0.051 | | 0.051 | |
| R-squared (between) | 0.915 | | 0.915 | | 0.922 | | 0.922 | |
| R-squared (Overall) | 0.546 | | 0.545 | | 0.596 | | 0.596 | |
| *Note*. CCI = Consumer Confidence Index, AVE = Asset Value Expectation. * *p* < 5%, ** *p* < 1%, *** *p* < 0.1%. Robust standard errors are in parentheses. CCI and AVE were indexed based on the formula from the Cabinet Office of Japan. | | | | | | | | |
