# Supplementary material for: Are consumer confidence and asset value expectations positively associated with length of daylight?: An exploration of psychological mediators between length of daylight and seasonal asset price transitions
Source: PLoS One. 2021 Jan 20;16(1):e0245520. doi: 10.1371/journal.pone.0245520 (PMC7817041; doi:10.1371/journal.pone.0245520)
Supplement: S6 Table — (DOCX) [file pone.0245520.s010.docx]

| **S6 Table. Fixed-effects model estimation of AVE with length of daylight, cloud cover, precipitation, and temperature (Model 3) for the two periods.** | | | | | | | | |
| --- | --- | --- | --- | --- | --- | --- | --- | --- |
|  | AVE until March 2011 | | AVE until March 2011 | | AVE after April 2011 | | AVE after April 2011 | |
| 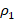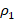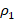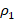   \|  \| \| --- \| | 0.025*** | (0.003) | 0.025*** | (0.003) | 0.075*** | (0.003) | 0.075*** | (0.003) |
| 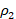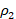   \|  \| \| --- \| | -0.027*** | (0.002) | -0.027*** | (0.002) | 0.018*** | (0.002) | 0.018*** | (0.002) |
| (per hour)*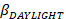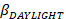* | 0.340*** | (0.015) | 0.296*** | (0.021) | 0.103*** | (0.013) | 0.284*** | (0.020) |
| (per one point)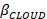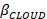   \| (per hour) \| \| --- \| |  |  | 0.059*** | (0.015) |  |  | -0.018 | (0.012) |
| *(per 1mm/day)*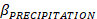   \| *(per 1mm/day)* \| \| --- \| |  |  | 0.002** | (0.001) |  |  | -0.001 | (0.001) |
| (per ℃)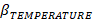   \| (per ℃) \| \| --- \| |  |  | 0.007 | (0.004) |  |  | -0.047*** | (0.004) |
| Intercept | 38.799*** | (0.267) | 38.786*** | (0.290) | 36.506*** | (0.221) | 35.211*** | (0.259) |
| No. of observations | 385,073 | | 385,073 | | 393,306 | | 393,306 | |
| No. of groups | 39,395 | | 39,395 | | 40,092 | | 40,092 | |
| R-squared (within) | 0.003 | | 0.003 | | 0.007 | | 0.007 | |
| R-squared (between) | 0.001 | | 0.001 | | 0.384 | | 0.373 | |
| R-squared (Overall) | 0.004 | | 0.003 | | 0.869 | | 0.857 | |

CCI = Consumer Confidence Index, AVE = Asset Value Expectation. ** *p* < 1%, *** *p* < 0.1%. Robust standard errors are in parentheses. CCI and AVE were indexed based on the formula from the Cabinet Office of Japan.
