# Supplementary material for: Are consumer confidence and asset value expectations positively associated with length of daylight?: An exploration of psychological mediators between length of daylight and seasonal asset price transitions
Source: PLoS One. 2021 Jan 20;16(1):e0245520. doi: 10.1371/journal.pone.0245520 (PMC7817041; doi:10.1371/journal.pone.0245520)
Supplement: S7 Table — (DOCX) [file pone.0245520.s011.docx]

| **S7 Table. Fixed-effects model to predict CCI and AVE from season and latitude (Model 4) for the two periods.** | | | | | | | | |
| --- | --- | --- | --- | --- | --- | --- | --- | --- |
|  | CCI until March 2011 | | CCI after April 2011 | | AVE until March 2011 | | AVE after April 2011 | |
| 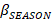   \|  \| \| --- \| | -0.0007 | (0.0055) | -0.0080 | (0.0052) | 0.0003 | (0.0064) | -0.0203** | (0.0063) |
| 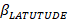   \|  \| \| --- \| | omitted | | omitted | | omitted | | omitted | |
| 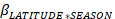   \|  \| \| --- \| | 0.0004* | (0.0002) | 0.0004** | (0.0001) | 0.0003 | (0.0002) | 0.0007*** | (0.0002) |
| Intercept | 41.7625*** | (0.0340) | 40.4433*** | (0.0315) | 41.7917*** | (0.0433) | 41.3248*** | (0.0399) |
| No. of observations | 472,727 | | 491,175 | | 472,727 | | 491,595 | |
| No. of groups | 44,654 | | 45,636 | | 44,654 | | 45,649 | |
| R-squared (within) | 0.0044 | | 0.0021 | | 0.0018 | | 0.0003 | |
| R-squared (between) | 0.0069 | | 0.0005 | | 0.0074 | | 0.0008 | |
| R-squared (overall) | 0.0026 | | 0.0004 | | 0.0016 | | 0.0000 | |
| *Note*. CCI = Consumer Confidence Index, AVE = Asset Value Expectation. * *p* < 5%, ** *p* < 1%, *** *p* < 0.1%. Robust standard errors are in parentheses. CCI and AVE were indexed based on the formula from the Cabinet Office of Japan. | | | | | | | | |
