# Supplementary material for: Are consumer confidence and asset value expectations positively associated with length of daylight?: An exploration of psychological mediators between length of daylight and seasonal asset price transitions
Source: PLoS One. 2021 Jan 20;16(1):e0245520. doi: 10.1371/journal.pone.0245520 (PMC7817041; doi:10.1371/journal.pone.0245520)
Supplement: S8 Table — (DOCX) [file pone.0245520.s012.docx]

| **S8 Table. The monthly summary statistics of CCI and AVE** **for the lower and higher latitude areas**. | | | | | | | | | | | | |
| --- | --- | --- | --- | --- | --- | --- | --- | --- | --- | --- | --- | --- |
|  | CCI in lower latitude areas | | | CCI in higher latitude areas | | | AVE in lower latitude areas | | | AVE in higher latitude areas | | |
|  | n | Mean | SD | n | Mean | SD | n | Mean | SD | n | Mean | SD |
| January | 38,985 | 41.77 | (15.09) | 38,226 | 41.91 | (15.12) | 39,001 | 42.47 | (17.85) | 38,241 | 42.61 | (18.11) |
| February | 39,113 | 41.52 | (15.12) | 38,291 | 41.71 | (15.14) | 39,126 | 41.68 | (17.95) | 38,304 | 41.93 | (18.11) |
| March | 38,975 | 41.50 | (15.16) | 37,956 | 41.80 | (15.25) | 38,990 | 41.93 | (19.01) | 37,971 | 42.18 | (19.37) |
| April | 42,969 | 41.79 | (14.96) | 41,958 | 42.09 | (15.14) | 43,010 | 42.49 | (17.97) | 41,997 | 42.83 | (18.10) |
| May | 43,018 | 42.73 | (14.55) | 41,935 | 43.11 | (14.61) | 43,041 | 43.00 | (17.24) | 41,965 | 43.46 | (17.43) |
| June | 42,538 | 42.31 | (14.67) | 41,614 | 42.55 | (14.77) | 42,564 | 42.38 | (18.55) | 41,641 | 42.79 | (18.68) |
| July | 42,552 | 42.60 | (14.43) | 41,691 | 42.98 | (14.47) | 42,562 | 42.52 | (17.06) | 41,703 | 43.08 | (17.21) |
| August | 42,460 | 42.58 | (14.32) | 41,495 | 42.81 | (14.39) | 42,477 | 42.33 | (17.00) | 41,501 | 42.79 | (17.18) |
| September | 39,307 | 42.09 | (14.57) | 38,312 | 42.34 | (14.67) | 39,319 | 41.80 | (18.41) | 38,331 | 41.86 | (18.66) |
| October | 39,406 | 41.85 | (14.53) | 38,402 | 41.92 | (14.62) | 39,419 | 41.64 | (17.49) | 38,422 | 42.01 | (17.70) |
| November | 39,302 | 41.44 | (14.83) | 38,299 | 41.45 | (14.99) | 39,316 | 41.57 | (17.83) | 38,308 | 41.86 | (17.94) |
| December | 38,975 | 40.53 | (15.42) | 38,123 | 40.58 | (15.51) | 38,984 | 41.01 | (19.19) | 38,129 | 41.17 | (19.38) |
| SD = Standard Deviation, CCI = Consumer Confidence Index, AVE = Asset Value Expectation | | | | | | | | | | | | |
