# Supplementary material for: Are consumer confidence and asset value expectations positively associated with length of daylight?: An exploration of psychological mediators between length of daylight and seasonal asset price transitions
Source: PLoS One. 2021 Jan 20;16(1):e0245520. doi: 10.1371/journal.pone.0245520 (PMC7817041; doi:10.1371/journal.pone.0245520)
Supplement: S9 Table — (DOCX) [file pone.0245520.s013.docx]

| **S9 Table. Fixed-effects model estimation of monthly CCI and AVE from Model 1 for the lower and higher latitude areas**. | | | | | | | | |
| --- | --- | --- | --- | --- | --- | --- | --- | --- |
|  | CCI in lower latitude areas | | CCI in higher latitude areas | | AVE in lower latitude areas | | AVE in higher latitude area | |
| 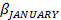   \|  \| \| --- \| | 1.118*** | (0.060) | 1.255*** | (0.061) | 1.345*** | (0.096) | 1.374*** | (0.098) |
| 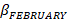   \|  \| \| --- \| | 0.791*** | (0.065) | 0.953*** | (0.065) | 0.494*** | (0.099) | 0.592*** | (0.102) |
| 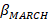   \|  \| \| --- \| | 1.001*** | (0.069) | 1.244*** | (0.070) | 0.937*** | (0.102) | 1.026*** | (0.104) |
| 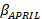   \|  \| \| --- \| | 0.866*** | (0.070) | 1.111*** | (0.072) | 1.105*** | (0.103) | 1.290*** | (0.104) |
| 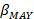   \|  \| \| --- \| | 1.810*** | (0.070) | 2.123*** | (0.072) | 1.649*** | (0.101) | 1.930*** | (0.103) |
| 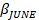   \|  \| \| --- \| | 1.570*** | (0.071) | 1.764*** | (0.072) | 1.159*** | (0.103) | 1.420*** | (0.105) |
| 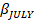   \|  \| \| --- \| | 1.811*** | (0.070) | 2.103*** | (0.071) | 1.290*** | (0.101) | 1.642*** | (0.103) |
| 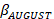   \|  \| \| --- \| | 1.800*** | (0.068) | 1.943*** | (0.070) | 1.129*** | (0.100) | 1.387*** | (0.101) |
| 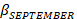   \|  \| \| --- \| | 1.557*** | (0.068) | 1.743*** | (0.069) | 0.733*** | (0.099) | 0.666*** | (0.103) |
| 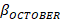   \|  \| \| --- \| | 1.273*** | (0.065) | 1.273*** | (0.066) | 0.574*** | (0.098) | 0.763*** | (0.101) |
| 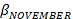   \|  \| \| --- \| | 0.777*** | (0.061) | 0.801*** | (0.062) | 0.418*** | (0.096) | 0.627*** | (0.097) |
| Intercept | 40.701*** | (0.047) | 40.751*** | (0.048) | 41.170*** | (0.071) | 41.326*** | (0.073) |
| No. of observations | 487,600 | | 476,302 | | 487,809 | | 476,513 | |
| No. of groups | 43,282 | | 42,458 | | 43,289 | | 42,464 | |
| R-squared (within) | 0.003 | | 0.004 | | 0.001 | | 0.002 | |
| R-squared (between) | 0.002 | | 0.002 | | 0.003 | | 0.003 | |
| R-squared (overall) | 0.002 | | 0.002 | | 0.001 | | 0.001 | |
| CCI = Consumer Confidence Index, AVE = Asset Value Expectation. *** *p* <0.1%. Robust standard errors are in parentheses. CCI and AVE were indexed based on the formula from the Cabinet Office of Japan. | | | | | | | | |
