# Supplementary material for: Are consumer confidence and asset value expectations positively associated with length of daylight?: An exploration of psychological mediators between length of daylight and seasonal asset price transitions
Source: PLoS One. 2021 Jan 20;16(1):e0245520. doi: 10.1371/journal.pone.0245520 (PMC7817041; doi:10.1371/journal.pone.0245520)
Supplement: S10 Table — (DOCX) [file pone.0245520.s014.docx]

| **S10 Table. Fixed-effects model estimation of CCI and AVE through cosinor model (Model 2) with amplitude and acrophase for the lower and higher latitude areas.** | | | | | | | | |
| --- | --- | --- | --- | --- | --- | --- | --- | --- |
|  | CCI in lower latitude areas | | CCI in higher latitude areas | | AVE in lower latitude areas | | AVE in higher latitude areas | |
| 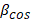   \|  \| \| --- \| | -0.618*** | (0.026) | -0.712*** | (0.027) | -0.406*** | (0.033) | -0.519*** | (0.033) |
| 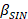   \|  \| \| --- \| | -0.094*** | (0.025) | -0.005 | (0.026) | 0.255*** | (0.033) | 0.270*** | (0.033) |
| Intercept | 41.899*** | (0.001) | 42.110*** | (0.001) | 42.073*** | (0.001) | 42.387*** | (0.001) |
| No. of observations | 487,600 | | 476,302 | | 487,809 | | 476,513 | |
| No. of groups | 43,282 | | 42,458 | | 43,289 | | 42,464 | |
| R-squared (within) | 0.002 | | 0.003 | | 0.001 | | 0.001 | |
| R-squared (between) | 0.002 | | 0.003 | | 0.003 | | 0.003 | |
| R-squared (overall) | 0.001 | | 0.002 | | 0.001 | | 0.001 | |
| Amplitude | 0.626 | (0.026) | 0.712 | (0.027) | 0.479 | (0.033) | 0.586 | (0.033) |
| Acrophase | 192.306 | (2.363) | 183.882 | (2.120) | 150.882 | (3.939) | 155.611 | (3.309) |
| CCI = Consumer Confidence Index, AVE = Asset Value Expectation. *** *p* < 0.1%. Robust standard errors are in parentheses except for amplitude and acrophase. For these, standard errors were calculated using the delta method. CCI and AVE were indexed based on the formula from the Cabinet Office of Japan. | | | | | | | | |
