# Supplementary material for: Are consumer confidence and asset value expectations positively associated with length of daylight?: An exploration of psychological mediators between length of daylight and seasonal asset price transitions
Source: PLoS One. 2021 Jan 20;16(1):e0245520. doi: 10.1371/journal.pone.0245520 (PMC7817041; doi:10.1371/journal.pone.0245520)
Supplement: S11 Table — (DOCX) [file pone.0245520.s015.docx]

| **S11 Table. Fixed-effects model estimation of CCI with length of daylight, cloud cover, precipitation, and temperature (Model 3) for the lower and higher latitude areas.** | | | | | | | | |
| --- | --- | --- | --- | --- | --- | --- | --- | --- |
|  | CCI in lower latitude areas | | CCI in lower latitude areas | | CCI in higher latitude areas | | CCI in higher latitude areas | |
| 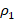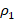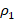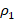   \|  \| \| --- \| | 0.201*** | (0.003) | 0.200*** | (0.003) | 0.205*** | (0.003) | 0.205*** | (0.003) |
| 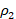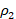   \|  \| \| --- \| | 0.060*** | (0.002) | 0.060*** | (0.002) | 0.060*** | (0.002) | 0.060*** | (0.002) |
| (per hour)*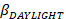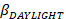* | 0.219*** | (0.010) | 0.165*** | (0.014) | 0.231*** | (0.009) | 0.219*** | (0.013) |
| (per one point)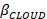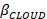   \| (per hour) \| \| --- \| |  |  | 0.074*** | (0.009) |  |  | 0.065*** | (0.009) |
| *(per 1mm/day)*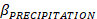   \| *(per 1mm/day)* \| \| --- \| |  |  | -0.001** | (0.000) |  |  | -0.001* | (0.001) |
| (per ℃)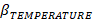   \| (per ℃) \| \| --- \| |  |  | 0.010*** | (0.003) |  |  | -0.002 | (0.003) |
| Intercept | 28.291*** | (0.191) | 28.348*** | (0.209) | 28.114*** | (0.183) | 27.855*** | (0.200) |
| No. of observations | 393,412 | | 393,412 | | 384,101 | | 384,101 | |
| No. of groups | 38,019 | | 38,019 | | 37,626 | | 37,626 | |
| R-squared (within) | 0.053 | | 0.054 | | 0.056 | | 0.056 | |
| R-squared (between) | 0.934 | | 0.934 | | 0.933 | | 0.933 | |
| R-squared (Overall) | 0.576 | | 0.575 | | 0.574 | | 0.574 | |
| CCI = Consumer Confidence Index, AVE = Asset Value Expectation. * *p* < 5%, **** *p* < 1%, ***** *p* < 0.1%. Robust standard errors are in parentheses. CCI and AVE were indexed based on the formula from the Cabinet Office of Japan. | | | | | | | | |
