# Supplementary material for: Are consumer confidence and asset value expectations positively associated with length of daylight?: An exploration of psychological mediators between length of daylight and seasonal asset price transitions
Source: PLoS One. 2021 Jan 20;16(1):e0245520. doi: 10.1371/journal.pone.0245520 (PMC7817041; doi:10.1371/journal.pone.0245520)
Supplement: S12 Table — (DOCX) [file pone.0245520.s016.docx]

| **S12 Table. Fixed-effects model estimation of AVE with length of daylight, cloud cover, precipitation, and temperature (Model 3) for the lower and higher latitude areas.** | | | | | | | | |
| --- | --- | --- | --- | --- | --- | --- | --- | --- |
|  | AVE in lower latitude areas | | AVE in lower latitude areas | | AVE in higher latitude areas | | AVE in higher latitude areas | |
| 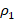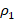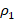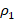   \|  \| \| --- \| |  | |  | |  | |  | |
|  | 0.053*** | (0.003) | 0.053*** | (0.003) | 0.058*** | (0.003) | 0.058*** | (0.003) |
| 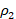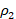   \|  \| \| --- \| | -0.003 | (0.002) | -0.003 | (0.002) | -0.001 | (0.002) | 0.000 | (0.002) |
| 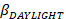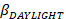(per hour) | 0.179*** | (0.015) | 0.273*** | (0.021) | 0.201*** | (0.013) | 0.280*** | (0.019) |
| \| (per one point) \| \| --- \|   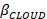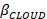 |  |  | 0.018 | (0.014) |  |  | 0.016 | (0.013) |
| \| 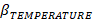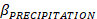*(per 1mm/day)* \| \| --- \| |  |  | -0.001 | (0.001) |  |  | 0.003*** | (0.001) |
| \| (per ℃) \| \| --- \| |  |  | -0.026*** | (0.004) |  |  | -0.026*** | (0.004) |
| Intercept | 37.770*** | (0.249) | 36.990*** | (0.282) | 37.532*** | (0.235) | 36.768*** | (0.265) |
| No. of observations | 393,833 | | 393,833 | | 384,546 | | 384,546 | |
| No. of groups | 38,026 | | 38,026 | | 37,634 | | 37,634 | |
| R-squared (within) | 0.004 | | 0.004 | | 0.004 | | 0.005 | |
| R-squared (between) | 0.854 | | 0.852 | | 0.846 | | 0.839 | |
| R-squared (Overall) | 0.256 | | 0.251 | | 0.263 | | 0.258 | |

CCI = Consumer Confidence Index, AVE = Asset Value Expectation. * *p* < 5%, ** *p* < 1%, *** *p* < 0.1%. Robust standard errors are in parentheses. CCI and AVE were indexed based on the formula from the Cabinet Office of Japan.
