# Supplementary material for: Are consumer confidence and asset value expectations positively associated with length of daylight?: An exploration of psychological mediators between length of daylight and seasonal asset price transitions
Source: PLoS One. 2021 Jan 20;16(1):e0245520. doi: 10.1371/journal.pone.0245520 (PMC7817041; doi:10.1371/journal.pone.0245520)
Supplement: S13 Table — (DOCX) [file pone.0245520.s017.docx]

| **S13 Table. Fixed-effects model to predict CCI and AVE from season and latitude (Model 4) for the lower and higher latitude areas.** | | | | | | | | |
| --- | --- | --- | --- | --- | --- | --- | --- | --- |
|  | CCI in lower latitude areas | | CCI in higher latitude areas | | AVE in lower latitude areas | | AVE in higher latitude areas | |
| 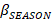 | -0.0188* | (0.0086) | 0.0066 | (0.0058) | -0.0152 | (0.0099) | -0.0031 | (0.0071) |
| 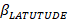 | omitted | | omitted | | omitted | | omitted | |
| 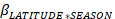 | 0.0008** | (0.0003) | 0.0001 | (0.0002) | 0.0006* | (0.0003) | 0.0003 | (0.0002) |
| Intercept | 41.1579*** | (0.0324) | 41.2392*** | (0.0331) | 41.5330*** | (0.0409) | 41.7025*** | (0.0419) |
| No. of observations | 487,600 | | 476,302 | | 487,809 | | 476,513 | |
| No. of groups | 43,282 | | 42,458 | | 43,289 | | 42,464 | |
| R-squared (within) | 0.0021 | | 0.0028 | | 0.0006 | | 0.0009 | |
| R-squared (between) | 0.0024 | | 0.0022 | | 0.001 | | 0.0006 | |
| R-squared (overall) | 0.0011 | | 0.0015 | | 0.0003 | | 0.0004 | |
| CCI = Consumer Confidence Index, AVE = Asset Value Expectation. * *p* < 5%, ** *p* < 1%, *** *p* < 0.1%. Robust standard errors are in parentheses. CCI and AVE were indexed based on the formula from the Cabinet Office of Japan. | | | | | | | | |
